# The grammar of interactive explanatory model analysis

**DOI:** 10.1007/s10618-023-00924-w

**Published:** 2023-02-14

**Authors:** Hubert Baniecki, Dariusz Parzych, Przemyslaw Biecek

**Affiliations:** 1 Warsaw University of Technology, Warsaw, Poland; 2 University of Warsaw, Warsaw, Poland

**Keywords:** Explainable AI, Model-agnostic explanation, Black-box model, Interactive explainability, Human-centered XAI

## Abstract

The growing need for in-depth analysis of predictive models leads to a series of new methods for explaining their local and global properties. Which of these methods is the best? It turns out that this is an ill-posed question. One cannot sufficiently explain a black-box machine learning model using a single method that gives only one perspective. Isolated explanations are prone to misunderstanding, leading to wrong or simplistic reasoning. This problem is known as the *Rashomon effect* and refers to diverse, even contradictory, interpretations of the same phenomenon. Surprisingly, most methods developed for explainable and responsible machine learning focus on a single-aspect of the model behavior. In contrast, we showcase the problem of explainability as an interactive and sequential analysis of a model. This paper proposes how different Explanatory Model Analysis (EMA) methods complement each other and discusses why it is essential to juxtapose them. The introduced process of Interactive EMA (IEMA) derives from the algorithmic side of explainable machine learning and aims to embrace ideas developed in cognitive sciences. We formalize the grammar of IEMA to describe human-model interaction. It is implemented in a widely used human-centered open-source software framework that adopts interactivity, customizability and automation as its main traits. We conduct a user study to evaluate the usefulness of IEMA, which indicates that an interactive sequential analysis of a model may increase the accuracy and confidence of human decision making.

## Introduction

Complex machine learning predictive models, often referred to as black-boxes, demonstrate high efficiency in a rapidly increasing number of applications. Simultaneously, there is a growing awareness among machine learning practitioners that we require more comprehensive tools for model interpretability and explainability. There are many technical discoveries in the field of explainable and interpretable machine learning (XIML) praised for their mathematical brilliance and software ingenuity (Baehrens et al. 2010; Ribeiro et al. 2016; Lundberg and Lee 2017; Biecek 2018; Alber et al. 2019; Apley and Zhu 2020). However, in all this rapid development, we forgot about how important is the interface between human and model. Working with models is highly interactive, so the data scientist’s tools should support this way of operation. Interactive interpreters, so-called REPL (read-eval-print-loop) environments, available in R or Python tools, significantly facilitated the data analysis process. Another breakthrough was notebooks that speed up the feedback loop in the model development process (Kluyver et al. 2016; Xie 2017). Not only is the process of building the model interactive, but, naturally, so is the process of analyzing and explaining the black-box. While Roscher et al. (2020) surveys XIML use for knowledge discovery, Lipton (2018) and Miller (2019) point out that there is a huge margin for improvement in the area of human-centered XIML.

People must trust models predictions to support their everyday life decisions and not harm them while doing so. Because of some spectacular black-box failures, even among the most technologically mature entities (Yu and Alì 2019; Rudin 2019), governments and unions step up to provide guidelines and regulations on machine learning decision systems to ensure their safeness, robustness and transparency (ACM US Public Policy Council 2017; European Commission 2020). The debate on the necessity of XIML is long over. With a *right to explanation* comes great responsibility for everyone creating algorithmic decision-making to deliver some form of proof that this decision is fair (Goodman and Flaxman 2017). Constructing and assessing such evidence becomes a troublesome and demanding task. Surprisingly we have a growing list of end-to-end frameworks for model development (Nguyen et al. 2019), yet not that many complete and convenient frameworks for model explainability.

We agree with Gill et al. (2020) that in practice, there are three main approaches to overcoming the opaqueness of black-box models: evading it and using algorithms interpretable by design (Rudin 2019), bias checking and applying mitigation techniques (Feldman et al. 2015), or using post-hoc explainability methods (Miller 2019). Although the first two are precise, the last solution is of particular interest to ours in this paper. We base our contribution on the philosophies of Exploratory Data Analysis (EDA) (Tukey 1977), which presents tools for in-depth data analysis, Explanatory Model Analysis (EMA) (Biecek and Burzykowski 2021), which presents tools for in-depth model analysis, and The Grammar of Graphics (Wilkinson 2005), which formalizes and unifies language for the visual description of data. Although the objective is set to bridge the research gap concerning opaque predictive models developed for *tabular data*, the introduced concept can be generalized to other tasks, specifically in deep learning.

*Objectives.* Wang et al. (2019) posits that we can extend XIML designs in many ways to embrace the human-centered approach to XIML, from which we distinguish the needs to (1) provide contrastive explanations that cross-compare different model’s aspects, (2) give exploratory information about the data that hides under the model in question and its explanations, (3) support the process with additional beneficial factors, e.g. explanation uncertainty, variable correlation, (4) integrate multiple explanations into a single, more cohesive dashboards. In this paper, we meet these objectives through a sequence of single-aspect explanations aiming to significantly extend our understanding of black-box models. Interactivity involves a sequence of operations; thus, explanatory model analysis can be seen as a cooperation between the operator and the explanatory interface. We adhere to the *Rashomon effect* (Breiman 2001) by juxtaposing complementary explanations, whereas conventionally it is used to denote analyzing diverging models.

*Contribution.* We formally define a language for human-model communication, which to our knowledge, is the first such work. The introduced grammar of Interactive Explanatory Model Analysis (IEMA) provides a multifaceted look at various possible explanations of the model’s behavior. We validate its usefulness in three real-world machine learning use-cases: an approachable and illustrative example based on the FIFA-20 regression task, an external model audit based on the COVID-19 classification task, and a user study based on the Acute Kidney Injury prediction task. This paper introduces and validates a methodology for which we already implemented and contributed an open-source software framework (Baniecki and Biecek 2019), as well as prototyped its applicability (Baniecki and Biecek 2021).

*Outline.* The paper is organized as follows. We start by discussing the related background and our previous work (Sect. 2). We introduce the grammar of IEMA that bases on a new taxonomy of explanations (Sect. 3) and present its applicability on two real-world predictive tasks (Sect. 4). We then report the results from a user study aiming to evaluate IEMA in a third practical setting (Sect. 5). Finally, we conclude with a discussion on the challenges in human-centered XIML (Sect. 6).

## Related work

### A theory-practice mismatch in explainable and interpretable machine learning

*Theory.* Research in cognitive sciences shows that there is a lot to be gained from the interdisciplinary look at XIML. Miller et al. (2017) and Miller (2019) continuously highlight that there is room for improvement in existing solutions, as most of them rarely take into account the human side of the black-box problem. While developing human-centered XIML frameworks, we should take into consideration the needs of multiple diverse stakeholders (Barredo Arrieta et al. 2020; Bhatt et al. 2020; Sokol and Flach 2020; Kuzba and Biecek 2020), which might require a thoughtful development of the user interface (Eiband et al. 2018). It is a different approach than in the case of machine learning frameworks, where we mostly care about the view of machine learning engineers. Hohman et al. (2018) comprehensively surveys research in the human-centered analysis of deep learning models. Srinivasan and Chander (2020) recommend further adoption of a human-centered approach in generating explanations, as well as understanding of the explanation context. Fürnkranz et al. (2020) perform user studies to analyze the plausibility of rule-based models that show that there is no negative correlation between the rule length and plausibility. We relate to these findings in proposing long sequences of explanations to analyze black-box models.

*Practice.* Focusing on overcoming the opacity in black-box machine learning has led to the development of various model-agnostic explanations (Friedman 2001; Ribeiro et al. 2016; Lundberg and Lee 2017; Lei et al. 2018; Fisher et al. 2019; Apley and Zhu 2020). There is a great need to condense many of those explanations into comprehensive frameworks for machine learning practitioners. Because of that, numerous technical solutions were born that aim to unify the programming language for model analysis (Biecek 2018; Alber et al. 2019; Greenwell and Boehmke 2020; Arya et al. 2020). They calculate various instance and model explanations, which help understand the model’s predictions next to its overall complex behavior. It is common practice to produce visualizations of these explanations as it might be more straightforward to interpret plots than raw numbers. Despite the unquestionable usefulness of the conventional XIML frameworks, they have a high entry threshold that requires programming proficiency and technical knowledge (Bhatt et al. 2020).

*Match.* We aim to (1) improve on the work related to more practical XIML methods, (2) satisfy the desideratum of the aforementioned theoretical contributions.

### Human-centered frameworks for explanatory model analysis

**Fig. 1:**
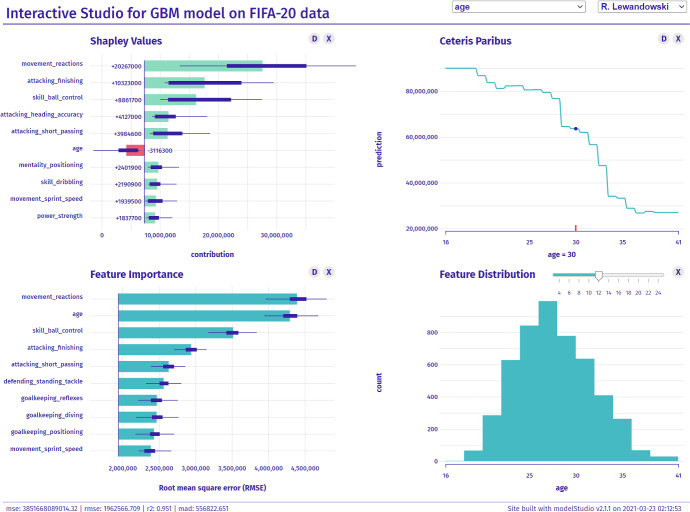
modelStudio automatically produces an HTML file—an interactive and customizable dashboard with model explanations and EDA visualizations. Here, we present a screenshot of its exemplary layout for the black-box model predicting a player’s value on the FIFA-20 data, see https://iema.drwhy.ai

In Baniecki and Biecek (2019), we introduced the modelStudio software package, which was a foundation for developing the grammar of IEMA introduced in this paper. modelStudio automatically computes various (data, instance and model) explanations and produces a customizable dashboard consisting of multiple panels for plots with their short descriptions. These are model-agnostic explanations and EDA visualizations. Such a serverless dashboard is easy to save, share and explore by all the interested parties. Interactive features allow for full customization of the visualization grid and productive model examination. Different views presented next to each other broaden the understanding of the path between the model’s inputs and outputs, which improves human interpretation of its decisions. Figure 1 presents an example of the modelStudio dashboard grid, which consists of complementary explanations–described in detail by Biecek and Burzykowski (2021). The key feature of the output produced with modelStudio is its interface, which is constructed to be user-friendly so that non-technical users have an easy time navigating through the process. There is a possibility to investigate a myriad of instances for local explanations at once by switching between them freely with a drop-down box. The same goes for all of the variables present in the model. Additionally, one can choose a custom grid of panels and change their position at any given time.

This solution puts a vast emphasis on implementing the grammar introduced in Sect. 3, performing IEMA like in Sect. 4, and overcoming the challenges discussed in Sect. 6. From our experience and the users’ feedback, working with the produced dashboard is engaging and effective. modelStudio lowers the entry threshold for all humans that want to understand the black-box predictive models. Due to the automated nature of dashboard generation, no sophisticated technical skills are required to produce it. Additionally, it shortens the human-model feedback loop in the machine learning development stage; thus, engineers may efficiently debug and improve models. Several tools relate to the modelStudio framework–we explicitly omit standard and well-established libraries for model interpretability and explainability as it is a widely documented ground (Adadi and Berrada 2018). As we further discuss in Sect. 6.2, they are not entirely going out towards emerging challenges. Although some ideas are discussed by Liu et al. (2017); Hohman et al. (2018), we are looking at tools that recently appeared in this area, especially new developments used in the machine learning practice. These are mostly interactive dashboard-like frameworks that focus on treating the model analysis as an extended process and take into account the human side of the black-box problem. Table 1 presents a brief comparison of relevant XIML frameworks. All of them take a step ahead to provide interactive dashboards with various complementary explanations that allow for a continuous model analysis process. Most of them produce such outputs automatically, which is a high convenience for the user. The ultimate XIML framework utilizes interactivity and customizability to suit different needs and scenarios.

**Table 1:** Comparison of the relevant XIML frameworks

	Instance explanation	Model explanation	EDA	Interactive	Automated	Customizable
modelStudio (Baniecki and Biecek 2019)	$\checkmark$	$\checkmark$	$\checkmark$	$\checkmark$	$\checkmark$	$\checkmark$
Driverless AI (Hall et al. 2019)	$\checkmark$	$\checkmark$	$\checkmark$	$\checkmark$	$\checkmark$	
InterpretML (Nori et al. 2019)	$\checkmark$	$\checkmark$	$\checkmark$	$\checkmark$		$\checkmark$
What-If Tool (Wexler et al. 2019)	$\checkmark$	$\checkmark$	$\checkmark$	$\checkmark$	$\checkmark$	$\checkmark$
Tensorboard (Google and Tang 2020)	$\checkmark$		$\checkmark$	$\checkmark$	$\checkmark$	
exBERT (Hoover et al. 2020)	$\checkmark$			$\checkmark$	$\checkmark$	
Arena (Piatyszek and Biecek 2021)	$\checkmark$	$\checkmark$	$\checkmark$	$\checkmark$	$\checkmark$	$\checkmark$
shapash (Golhen et al. 2021)	$\checkmark$	$\checkmark$	$\checkmark$	$\checkmark$	$\checkmark$	

Interactive, customizable, and automated tools become more approachable for diverse stakeholders, apparent in the XIML domain

Driverless AI (Hall et al. 2019) is a comprehensive state-of-the-art commercial machine learning platform. It automates variable engineering, model building, visualization, and explainability. The last module supports some of the instance and model explanations and, most importantly, does not require the user to know how to produce them. The framework also delivers documentation that describes the complex explainable machine learning nuances. The main disadvantages of this framework are its commercial nature and lack of customization options. InterpretML (Nori et al. 2019) provides a unified API for model analysis. It can be used to produce explanations for both white-box and black-box models. The ability to create a fully customizable interactive dashboard, that also compares many models at the same time, is a crucial advantage of this tool. Unfortunately, it does not support automation, which, especially for inexperienced people, could be a helpful addition to such a complete package. TensorBoard (Google and Tang 2020) is a dashboard that visualizes model behavior from various angles. It allows tracking models structure, project embeddings to a lower-dimensional space or display audio, image and text data. More related is the What-If Tool (Wexler et al. 2019) that allows machine learning engineers to explain algorithmic decision-making systems with minimal coding. Using it to join all the metrics and plots into a single, interactive dashboard embraces the grammar of IEMA. What differentiates it from modelStudio is its sophisticated user interface that becomes a barrier for non-technical users. explAIner (Spinner et al. 2019) is similar to What-If Tool adaptation of the TensorBoard dashboard. It focuses on explainable and interactive machine learning, contributing a more conceptual framework to perform user-studies on these topics. exBERT (Hoover et al. 2020) is an interactive tool that aims to explain the state-of-the-art Natural Language Processing (NLP) model BERT. It enables users to explore what and how transformers learn to model languages. It is possible to input any sentence which is then parsed into tokens and passed through the model. The attentions and ensuing word embeddings of each encoder are extracted and displayed for interaction. This shows a different proposition adapted for the NLP use case but still possesses key traits like automation and interactivity of the dashboard. Finally, the most recent software contributions are Arena (Piatyszek and Biecek 2021) and shapash (Golhen et al. 2021).

Overall, the human-centered frameworks used for explanatory model analysis reflect the ideas of juxtaposing complementary explanations and IEMA, which further motivates us to define the grammar.

### Evaluating interactive explanations in user studies

In Sect. 5, we conduct a user study with human participants with the aim of evaluating the grammar of IEMA. Historically, evaluation with human subjects involved asking laypeople to choose a better model based on its explanations, and following it with general questions about model trust (Ribeiro et al. 2016). More recently, various ways of evaluating explanations are considered, e.g. conducting technical experiments that resemble measures based on heuristically defined notions of explainability (Vilone and Longo 2021). Nevertheless, the fundamental approach is to evaluate explanations from the perspective of the end-users, for example, by asking them questions on the explanations’ quality with answers based on a well-established Likert scale (Hoffman et al. 2018). In this manner, Adebayo et al. (2020) assess the users’ ability to identify bugged models relying on wrong data signals based on explanations. Samuel et al. (2021) evaluates the predictability, consistency, and reliability of saliency-based explanations. Mishra and Rzeszotarski (2021) evaluates how concept-based explanations improve the users’ performance in estimating the model’s predictions and confidence. Similarly, Poursabzi-Sangdeh et al. (2021) evaluates how interpretability in models improves the users’ performance in estimating predictions based on a numeric interval scale. In all of these studies, participants provided answers based on a single explanation (set) for a specific data point or image. On the contrary, our study aims to evaluate an *interactive sequential* model analysis process.

There were a few attempts to quantify such a process. Jesus et al. (2021) consider a real-world fraud detection task and gradually increase the information provided to the participants in three stages: data only, data with the model’s prediction, and data with the model’s prediction and its explanation. The last step quantifies the general impact of explainability on human performance, and the study results in a conclusion that the tested explanations improve human accuracy. In our case, the baseline consists of data with a prediction and a single explanation, and we gradually increase the information in the form of juxtaposing complementary explanations. The closest to our work is the i-Algebra framework—an interactive language for explaining neural networks (Zhang et al. 2021). It is evaluated in three human case studies, like inspecting adversarial inputs and resolving inconsistency between models. Although the introduced SQL-like language considers interactively querying various explanation aspects, in a study, participants were asked to use only one specific query to answer a given question. Our user study puts more emphasis on comparing multiple explanation aspects, specifically for tabular data.

On a final note, one can conduct a study on a targeted group of participants, e.g. machine learning or domain experts (Samuel et al. 2021; Jesus et al. 2021), or through crowd-sourced experiments with more random users on platforms like Amazon Mechanical Turk (MTurk) (Ribeiro et al. 2016; Mishra and Rzeszotarski 2021; Poursabzi-Sangdeh et al. 2021; Zhang et al. 2021). Oftenly this is a quality-quantity trade-off since experts’ answers may be of higher quality, but such participants are a scarce resource. We omit using MTurk and target machine learning experts, focusing on a higher quality over the number of answers.

## The grammar of interactive explanatory model analysis

**Fig. 2:**
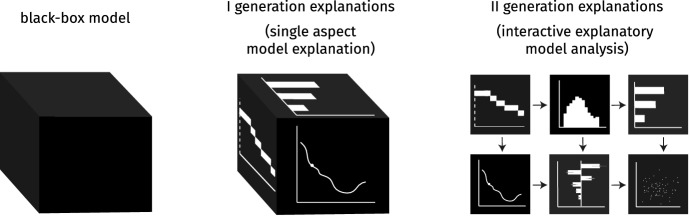
Increasing computing power and the availability of automated machine learning tools resulted in complex models that are effectively black-boxes. The first generation of model explanations aims at exploring individual aspects of model behavior. The second generation of model explanation aims to integrate individual aspects into a vibrant and multi-threaded customizable story about the black-box that addresses the needs of various stakeholders. We call this process Interactive Explanatory Model Analysis (IEMA)

Figure 2 shows how the perception of black-box machine learning changes with time. For some time, model transparency was not considered necessary, and the main focus was put on model performance. The next step was the first generation of explanations focused on individual model’s aspects, e.g. the effects and importances of particular variables. The next generation focuses on the analysis of various model’s aspects. The second generation’s requirements involve a well-defined taxonomy of explanations and a definition of the grammar generating their sequences. We first introduce a new taxonomy of methods for model analysis, and then, on its basis, we formalize the grammar of IEMA to show how different methods complement each other.

### Taxonomy of explanations in IEMA

**Fig. 3:**
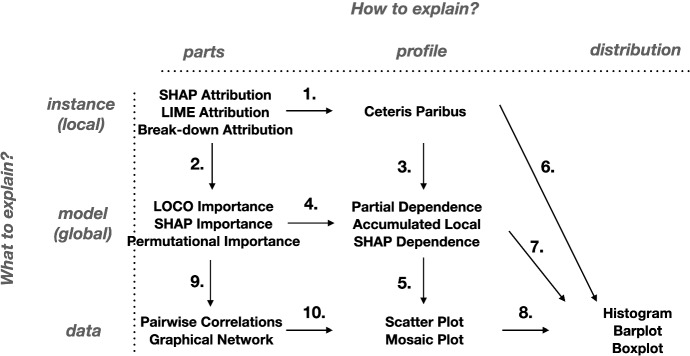
The concept of Interactive Explanatory Model Analysis shows how the various methods for model analysis enrich each other. Columns and rows span the taxonomy of explanations in IEMA, where names of well-known techniques are listed in cells. The graph’s edges indicate complementary explanations

The taxonomy of explanations in IEMA consists of two dimensions presented in Fig. 3. It is based on EMA (Biecek and Burzykowski 2021) and accordant with the alternative XIML taxonomies (Molnar 2020; Lundberg et al. 2020; Barredo Arrieta et al. 2020; Arya et al. 2020). The first dimension categorizes single-aspect explanations with respect to the question “What to explain?”. The second dimension groups the methods with respect to the question “How to explain?”. The proposed taxonomy distinguishes three key objects answering the “What to explain?” question.

1. **Data exploration** techniques have the longest history, see EDA (Tukey 1977). They focus on the presentation of the distribution of individual variables or relationships between pairs of variables. Often EDA is conducted to identify outliers or abnormal instances; it may be interesting to every stakeholder, but most important is for model developers. Understanding data allows them to build better models. For semantic reasons and clarity in the grammar of IEMA, we further relate to these methods as *data explanations*.
2. **Global model explanation** techniques focus on the model’s behaviour on a certain dataset. Unlike data explanations, the main focus is put on a particular model. We could have many differing models for one dataset, i.e. in the number of variables. Various stakeholders use global methods, but they are often of interest to model validators, which check whether a model behaves as expected. Examples of such methods are: model performance metrics, SHapley Additive exPlanations (SHAP) (Lundberg and Lee 2017), Permutational Importance (Fisher et al. 2019; Greenwell and Boehmke 2020), Partial Dependence Plots (PDP) (Friedman 2001; Greenwell 2017), Accumulated Local Effects (ALE) (Apley and Zhu 2020).
3. **Local instance explanation** techniques deal with the model’s output for a single instance. This type of analysis is useful for detailed model debugging, but also to justify the decision proposed by the model to the end-users. Examples of such methods are: LIME (Ribeiro et al. 2016), SHAP (Lundberg and Lee 2017), Break-down Attribution (Staniak and Biecek 2018), Ceteris Paribus (CP) (Biecek and Burzykowski 2021).

The second dimension groups the explainability methods based on the nature of the performed analysis. Similarly, we distinguish three types here.

1. **Analysis of parts** focuses on the importance of the model’s components—single variables or groups of variables. The model’s output can be quantified by evaluating its quality or average prediction. Examples of such methods are: LOCO (Lei et al. 2018), LIME, Break-down, SHAP, Permutational Importance.
2. **Analysis of the profile** covers the effect of a target variable to changes in an explanatory variable. The typical result is a prediction profile as a function of the selected variable in the input data. Examples of such methods are: CP, PDP, ALE.
3. **Analysis of the distribution** shows the distribution of certain variables in the data. The results make it easier to understand how typical are certain values.

Figure 3 shows how EMA techniques fit the proposed taxonomy. These are 17 methods for explaining data, models and instances. The list might not be exhaustive, and more methods to explain particular aspects of the model will certainly be developed over time. We refer to the appropriate papers and books for explanations’ definitions, as we focus on providing a level of abstraction over the well-known methods used in XIML practice. Nevertheless, we introduce the following notation to strengthen the intuition.

Global explanations operate on a dataset and a model. Let $X^{\;n\times p}$ stand for a dataset with *n* rows and *p* columns. Here *p* stands for the number of variables while *n* stands for the number of instances. Let $f: {\mathcal {X}} \rightarrow {\mathcal {R}}$ denote for the model of interest, where ${\mathcal {X}} = {\mathcal {R}}^{p}$ is the *p*-dimensional input space. Local explanations additionally operate on a single instance. Let $x^* \in {\mathcal {X}}$ stand for the instance of interest; often $x^*$ is an observation from *X*.

When we refer to the analysis of an *instance profile*, we are interested in a function that summarises how the model *f* responds to changes in variable $X_j$. For local explanations such as CP, the profile *g*(*z*) for variable $X_j$ and instance $x^*$ is defined as

1 $$\begin{aligned} g_{x^*_j}(z) = f(x^*|x^*_j=z), \end{aligned}$$

where $\;x^*_j\;=\;z\;$ means that the value of variable $X_j$ in an instance $x^*$ is changed to *z*. When we refer to the analysis of *instance parts*, we are interested in the attribution of individual variables to some measure. For local explanations such as SHAP Attribution, we want the variable attributions $h(x^*_j)$ of variables $X_j$ that sum up to a model prediction for an instance $x^*$

2 $$\begin{aligned} \sum _{j=1}^p h(x^*_j) = f(x^*). \end{aligned}$$

Global explanations may be defined as some aggregation of the local explanations, e.g. over the whole dataset. For *model profile* explanations like PDP, *G*(*z*) is an average of CP over all instances $x^i \in X$

3 $$\begin{aligned} G_{X_j}(z) = \frac{1}{n} \sum _{i=1}^n g_{x^i_j}(z). \end{aligned}$$

For *model parts* explanations like SHAP Importance, $H_X(X_j)$ is an average of absolute SHAP Attribution values over all instances $x^i \in X$

4 $$\begin{aligned} H_X(X_j) = \frac{1}{n} \sum _{i=1}^n \left| h(x_j^i)\right| . \end{aligned}$$

### Context-free grammar of IEMA

In the previous section, we described the intuition behind the IEMA grammar. However, to be able to generate explanations, we need a formalised notation of this concept. In this section, we define the context-free grammar of IEMA to generate a language of explanations’ sequences (Chomsky 1956). A context-free grammar *G* is defined by the 4-tuple $G=(N,\;T,\;R,\;S)$, where:

- *N* is a set of nonterminal symbols which correspond to the concepts in taxonomy of IEMA (Fig. 3). These have names with only lowercase letters in Table 2, e.g. model_explanation, model_parts_.
- *T* is a set of terminal symbols that correspond to the data, instance, and model explanations. These have names with uppercase letters in Table 3, e.g. Histogram.
- *R* is a set of rules denoted with $\rightarrow$ and $\mid$ in Tables 2 and 3.
- *S* is the start symbol denoted as explanation in Table 2.

Finally, $\varepsilon$ stands for the *NULL* symbol. The presented rules are a formal way of understanding the grammar of IEMA. These allow for defining the process of explanatory model analysis, which in practice becomes an interactive and sequential analysis of a model that utilizes human-centered frameworks.

**Table 2:** Rules defining the context-free grammar of IEMA

explanation	$\rightarrow$	instance_explanation $\ \ \ \mid$
		model_explanation $\ \ \ \mid$
		data_explanation
instance_explanation	$\rightarrow$	instance_parts $\cdot$ instance_parts_
instance_parts_	$\rightarrow$	Select_Variable $\cdot$ instance_profile $\cdot$ instance_profile_ $\cdot$ instance_parts_ $\ \ \ \mid$
		model_parts $\cdot$ model_parts_ $\cdot$ instance_parts_ $\ \ \ \mid$
		$$\varepsilon $$
instance_profile_	$\rightarrow$	data_distribution $\cdot$ instance_profile_ $\ \ \ \mid$
		model_profile $\cdot$ model_profile_ $\cdot$ instance_profile_ $\ \ \ \mid$
		$$\varepsilon $$
model_explanation	$\rightarrow$	model_parts $\cdot$ model_parts_
model_parts_	$\rightarrow$	Select_Variable $\cdot$ model_profile $\cdot$ model_profile_ $\cdot$ model_parts_ $\ \ \ \mid$
		data_parts $\cdot$ data_parts_ $\cdot$ model_parts_ $\ \ \ \mid$
		$$\varepsilon $$
model_profile_	$\rightarrow$	data_profile $\cdot$ data_profile_$\cdot$ model_profile_ $\ \ \ \mid$
		data_distribution $\cdot$ model_profile_ $\ \ \ \mid$
		$$\varepsilon $$
data_explanation	$\rightarrow$	data_parts $\cdot$ data_parts_ $\ \ \ \mid$
		$$\varepsilon $$
data_parts_	$\rightarrow$	data_profile $\cdot$ data_profile_ $\ \ \ \mid$
		$$\varepsilon $$
data_profile_	$\rightarrow$	data_profile $\cdot$ data_parts_ $\ \ \ \mid$
		data_distribution $\ \ \ \mid$
		$$\varepsilon $$

These start with nonterminal symbols; most notably explanation is the start symbol

**Table 3:** Representation of possible terminal symbols in the context-free grammar of IEMA

data_parts	$\rightarrow$	Pairwise_Correlation $\ \ \ \mid$
		Graphical_Networks
data_profile	$\rightarrow$	Scatter_Plot $\ \ \ \mid$
		Mosaic_Plot
data_distribution	$\rightarrow$	Histogram $\ \ \ \mid$
		Boxplot $\ \ \ \mid$
		Barplot
model_parts	$\rightarrow$	Permutational_Importance $\ \ \ \mid$
		LOCO_Importance $\ \ \ \mid$
		SHAP_Importance
model_profile	$\rightarrow$	Partial_Dependence $\ \ \ \mid$
		Accumulated_Local $\ \ \ \mid$
		SHAP_Dependence
instance_parts	$\rightarrow$	SHAP_Attribution $\ \ \ \mid$
		BD_Attribution $\ \ \ \mid$
		LIME_Attribution
instance_profile	$\rightarrow$	Ceteris_Paribus

These correspond to the taxonomy of explanations

### Complementary explanations in IEMA

The explanatory techniques presented in Fig. 3 are focused on explaining only a single perspective of the instance, model or data; hence, these enhance our understanding of the black-box only partially. The main results of this paper are based on the observation that each explanation generates further cognitive questions. EMA adds up to chains of questions joined with explanations of different types. *Juxtapositioning* of different explanations helps us to understand the model’s behavior itself better. Novel XIML techniques aim to provide various complementary perspectives because EMA is a process in which answering one question raises new ones. The introduced approach implies designing a flexible, interactive system for EMA in which we plan possible paths between the model’s perspectives that complement each other.

**Fig. 4:**
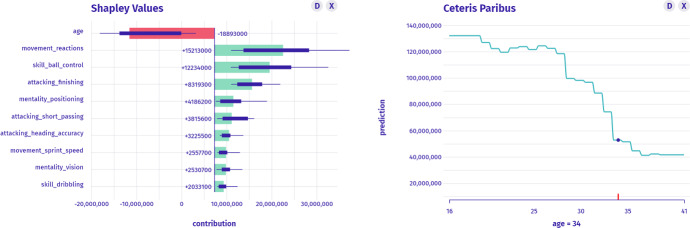
Left: SHAP Attributions to the model’s prediction shows which variables are most important for a specific instance. Right: Ceteris Paribus shows the instance prediction profile for a specific variable

**Fig. 5:**
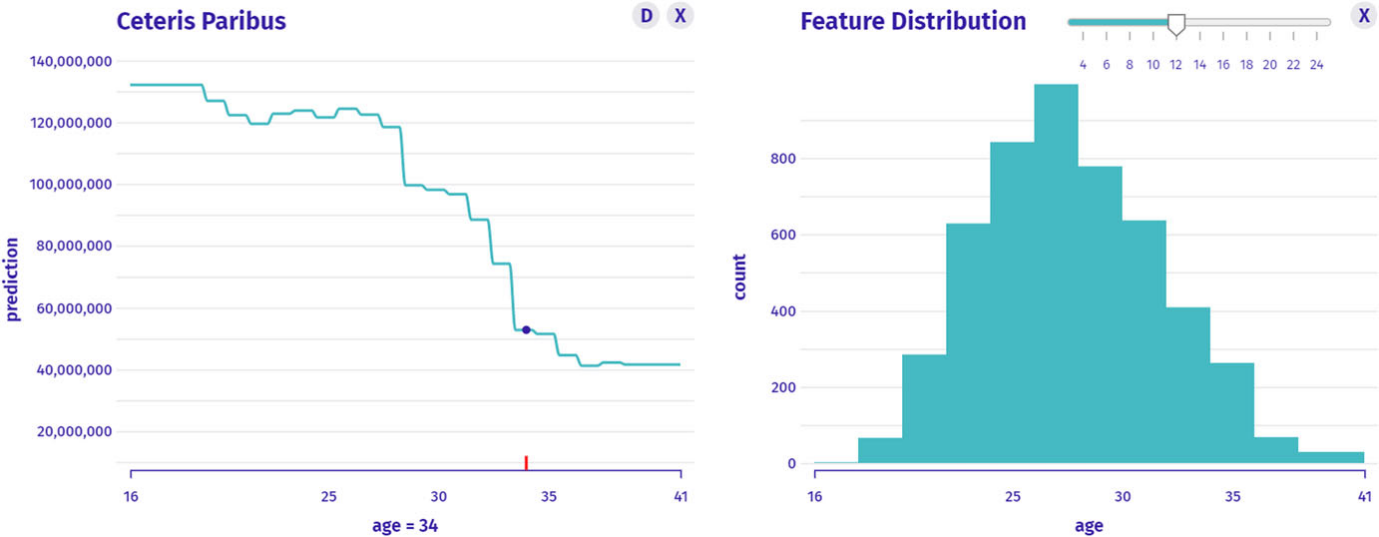
Left: Ceteris Paribus for the age variable shows the monotonicity of the instance prediction profile, for which values are large or small. Right: Histogram shows the distribution of the age variable’s values

**Fig. 6:**
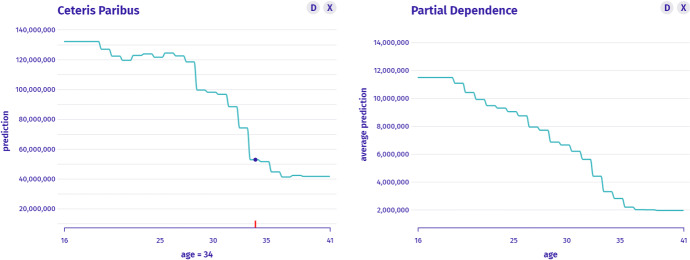
Left: Ceteris Paribus for a single instance shows how the model behaves in its neighbourhood. Right: Partial Dependence shows an average model prediction profile that agrees with instance analysis

We define interactions with the machine learning system as a set of possible paths between these complementary explanations. Figure 3 shows a proposed graph of interactions, which creates the grammar of IEMA. The edge in the graph denotes that the selected two explanations complement each other. For example Fig. 4 shows an interaction for edge 1, Fig. 5 shows an interaction for edge 6, while Fig. 6 shows an interaction for edge 3.

## Exemplary use-cases

We have already introduced the taxonomy of explanations and the grammar of IEMA. Now, we present these XIML developments based on two predictive tasks.

### Regression task of predicting the FIFA-20 player’s value

*Setup* In the first use-case, we apply the grammar of IEMA to the Gradient Boosting Machine (Friedman 2001) model predicting player’s value based on the FIFA-20 dataset (Leone 2020). We aim to show a universal example of knowledge discovery with explainable machine learning. We only use model-agnostic explanations; thus, the model’s structure is irrelevant—we refer to it as a *black-box* model. We construct the sequence of questions using the introduced grammar to provide a broad understanding of the black-box. We start with an analysis of the model’s prediction for a single instance, more precisely Cristiano Ronaldo’s (CR).^1^ The black-box model estimates CR’s value at 38M Euro. Consider the following human-model interaction *I*:

$I_1$: *What factors have the greatest influence on the estimation of the worth of Cristiano Ronaldo?* In the taxonomy, this is the instance-level question about parts. To answer this question, we may present SHAP or Break-down Attributions as in Fig. 4. The movement_reactions and skill_ball_control variable increases worth the most, while the age is the only variable that decreases CR’s worth.

$I_2$: *What is the relationship between age and the worth of CR? What would the valuation be if CR was younger or older?* This is an instance-level question about the profile which we answer with the Ceteris Paribus technique in Fig. 5. Between the extreme values of the age variable, the player’s worth differs more than three times.

$I_3$: *How many players are Cristiano Ronaldo’s age?* In the taxonomy, this is a model-level question about the distribution. Histogram answers the question as presented in Fig. 5. We see that the vast majority of players in the data are younger than CR; thus, his neighbourhood might not be well estimated by the model.

$I_4$: *Whether such relation between age and worth is typical for other players?* This is a model-level question about the profile that we answer with Partial Dependence as presented in Fig. 6. We see a global pattern that age reduces the player’s worth about five times (with established skills). However, we suspect that younger players have lower skills, so another question arises.

**Fig. 7:**
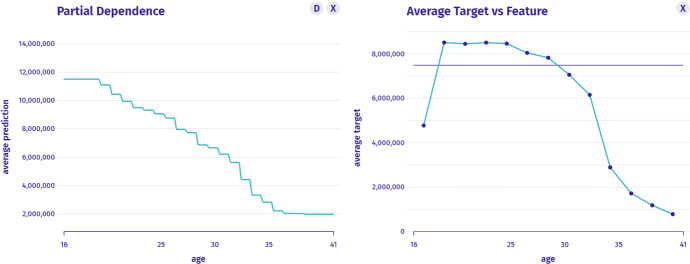
Left: Partial Dependence shows the explanation of average model’s prediction. Right: The average value of the target variable as a function of the selected variable shows the data explanation for comparison

$I_5$: *What is the relationship between the valuation and age in the original data?* This is the data-level question about the profile answered by Fig. 7. Finally, we might ask more questions concerning the overall model’s behavior.

**Fig. 8:**
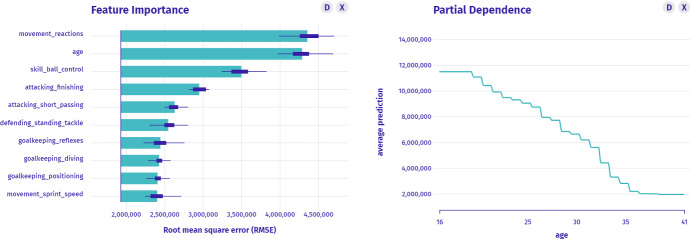
Left: Permutational Importance shows which variables influence the model prediction the most. Right: Partial Dependence may imply high variable importance by the model profile variability

$I_6$: *Which variables are the most important when all players are taken into account?* In the introduced taxonomy, this is a model-level question about the parts answered by Fig. 8. There are three: movement_reactions, age and skill_ball_control variables are the most important to the black-box model with high certainty.

**Fig. 9:**
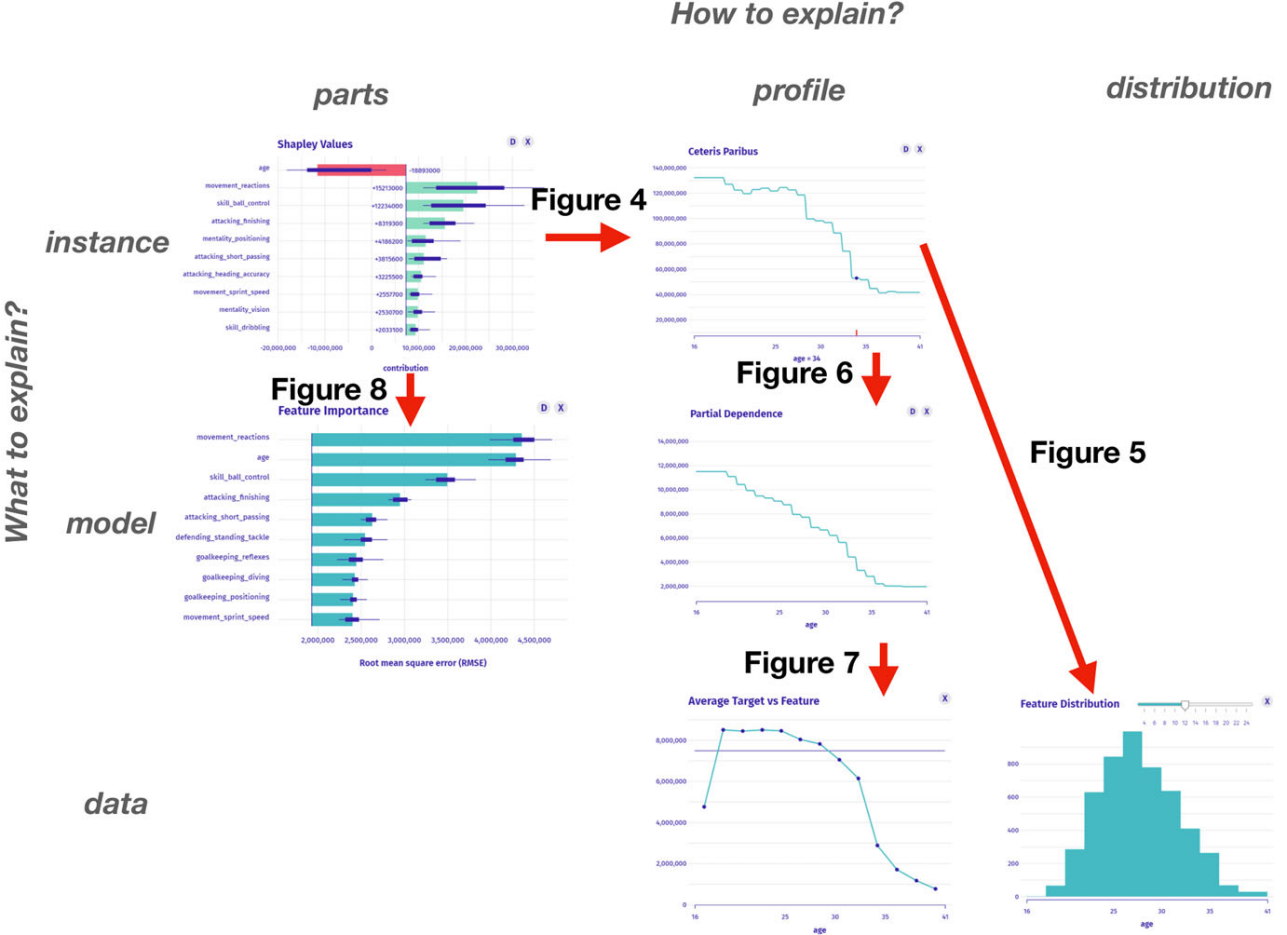
Summary of a single path in the Interactive Explanatory Model Analysis of FIFA-20 use-case. Different users may choose different orders to explore this graph using the introduced grammar of IEMA

**Fig. 10:**
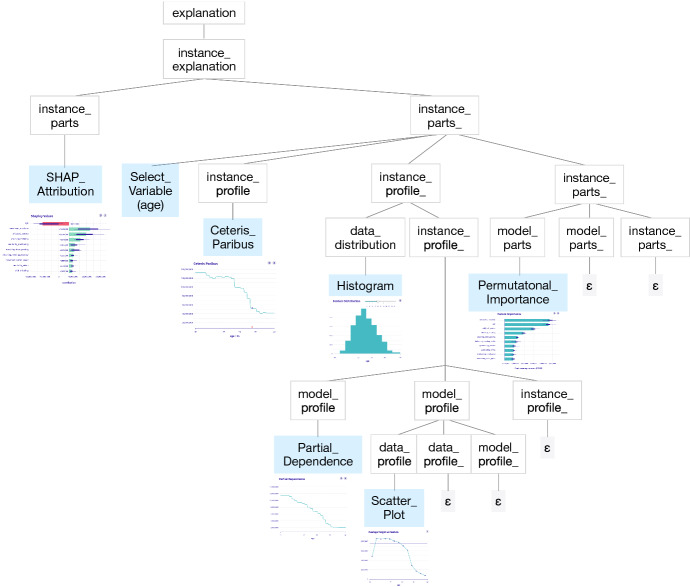
Parsing tree for the example from Fig. 9. It represents the semantic information that symbols derived from the grammar of IEMA. Blue leaves indicate the terminal symbols, e.g. XIML methods (Color figure online)

$I_1$–$I_6$: *A human-model interaction.* Figures 4, 5, 6, 7 and 8 show the process of interactive explanatory model analysis. No single explanation gives as much information about the model as the sequence of various model’s aspects. The grammar of IEMA allows for the prior calculation of potential paths between explanations summarised in Fig. 9. To keep the thoughts flowing, the desired tool must provide interactive features, customizability and ensure a quick feedback-loop between questions. These functionalities are available^2^ in the open-source modelStudio package (Baniecki and Biecek 2019) and partially other human-centered frameworks, which we briefly preview in Sect. 2.2. Figure 10 shows the parsing tree for the presented exemplary path.

### Classification task of predicting the COVID-19 patient’s mortality

In the medicine domain, machine learning supporting knowledge discovery and decision-making becomes more popular. Historically, interpretable white-box models were used to facilitate both of these tasks, as they provide transparent prediction attributions and variable importances (Rudin 2019). Nowadays, black-boxes may provide better performance and robust generalization, but there is a vital necessity to explain their behavior; thus, XIML is a crucial factor in various predictive tasks concerning medical data (Lundberg et al. 2020; Bruckert et al. 2020). Schmid and Finzel (2020) showcase a system for human-model interaction that supports decision-making with a deep learning black-box in medicine.

Contrastively, Yan et al. (2020) create an interpretable decision tree that supports decision-making in a hospital, concerning COVID-19 mortality. We applied the methodology of IEMA to showcase the potential human-model interaction with a machine learning black-box in this use-case (Baniecki and Biecek 2021). It results in a list of potential questions that appear in explanatory model analysis and a practical tool that could be used in place of a standard decision tree.^3^ The grammar of IEMA becomes useful in the external audit of machine learning models.

## Evaluation with human subjects: a user study

We conduct a user study on 30 human subjects to evaluate the usefulness and need for IEMA in a real-world setting. The goal is to assess if an interactive and sequential analysis of a model brings value to explaining black-box machine learning. In that, we aim to answer the main hypothesis of “Juxtaposing complementary explanations increases the usefulness of explanations.” The *usefulness* can be measured in varied ways; in this case, we aim to check if juxtaposing complementary explanations *increases*:

- $H_1$: human *accuracy* in understanding the model,
- $H_2$: human *confidence* in understanding the model.

The latter can alternatively be viewed as increasing *trust* in machine learning models.

*Task description.* We chose a binary classification task from a medical domain for this study. It considers an authentic machine learning use case: predicting the occurrence of Acute Kidney Injury (AKI) in patients hospitalized with COVID-19. Physicians aim to estimate the probability of AKI based on the patient’s blood test and medical history. Model engineers are tasked with developing and auditing a random forest algorithm for supporting such decisions. Overall, practitioners aim to use model explanations to allow for meaningful interpretation of its predictions. Let’s consider a scenario in which, before deploying the model, a developer performs its audit by examining predictions with their explanations. Part of this audit is to look for wrong model behaviour based on abnormalities in either one. We aim to analyze how juxtaposing complementary explanations affect human accuracy and confidence in finding wrong model predictions.

*Experimental setting.* In this study, we rely on data of 390 patients from the clinical department of internal diseases in one of the Polish hospitals. The original values were altered slightly to maintain their anonymity. For each patient, we have information about 12 variables determined during the patient’s admission: two quantitative variables that are biomarkers from a blood test: creatinine and myoglobin, five binary variables indicating chronic diseases: hypertension (among 62% of patients), diabetes (28%), cardiac atherosclerosis (19%), hyperlipidemia (32%), chronic kidney disease (5%); and five binary variables indicating symptoms related to COVID-19: fever (among 82% of patients), respiratory problems (90%), digestive problems (26%), neurological problems (8%), a critical condition requiring ventilator (6%). The classified target variable is a relatively rare binary variable: an occurrence of AKI during the patient’s hospitalization (among 18% of patients). Overall, the above-described structure of the data was designed to be easily comprehended by the participants of our user study. There are two critical continuous variables, and the remaining binary ones can additionally affect the predicted outcome. Based on the data, we trained a random forest model with 100 trees and a tree depth of 3 for predicting AKI, which is treated as a black-box, later with an intention to deploy it in a hospital. To balance the training process, patients were weighted by the target outcome, therefore the model returns a rather uniformly-distributed probability of AKI (a number between 0 and 1). Assuming a classification threshold of 0.5, it achieved the following binary classification measures: Accuracy (0.896), AUC (0.946), F1 (0.739), Precision (0.644), Recall (0.866), which is more than needed for our user study.

*Questionnaire description.* We design a user study as an about 45-min questionnaire, in which each participant is tasked with sequentially auditing the predictions with explanations for 12 patients. Specifically, a participant is asked to answer the question “Is the class predicted by the model for this patient accurate?” based on:

1. a single Break-down explanation ($Q_1$),
2. the same Break-down explanation with an additional Ceteris Paribus explanation of the most important variable based on the highest value in Break-down ($Q_2$),
3. the above-mentioned set of explanations with an additional Shapley Values explanation and a Ceteris Paribus explanation of an arbitrarily chosen variable ($Q_3$).

**Fig. 11:**
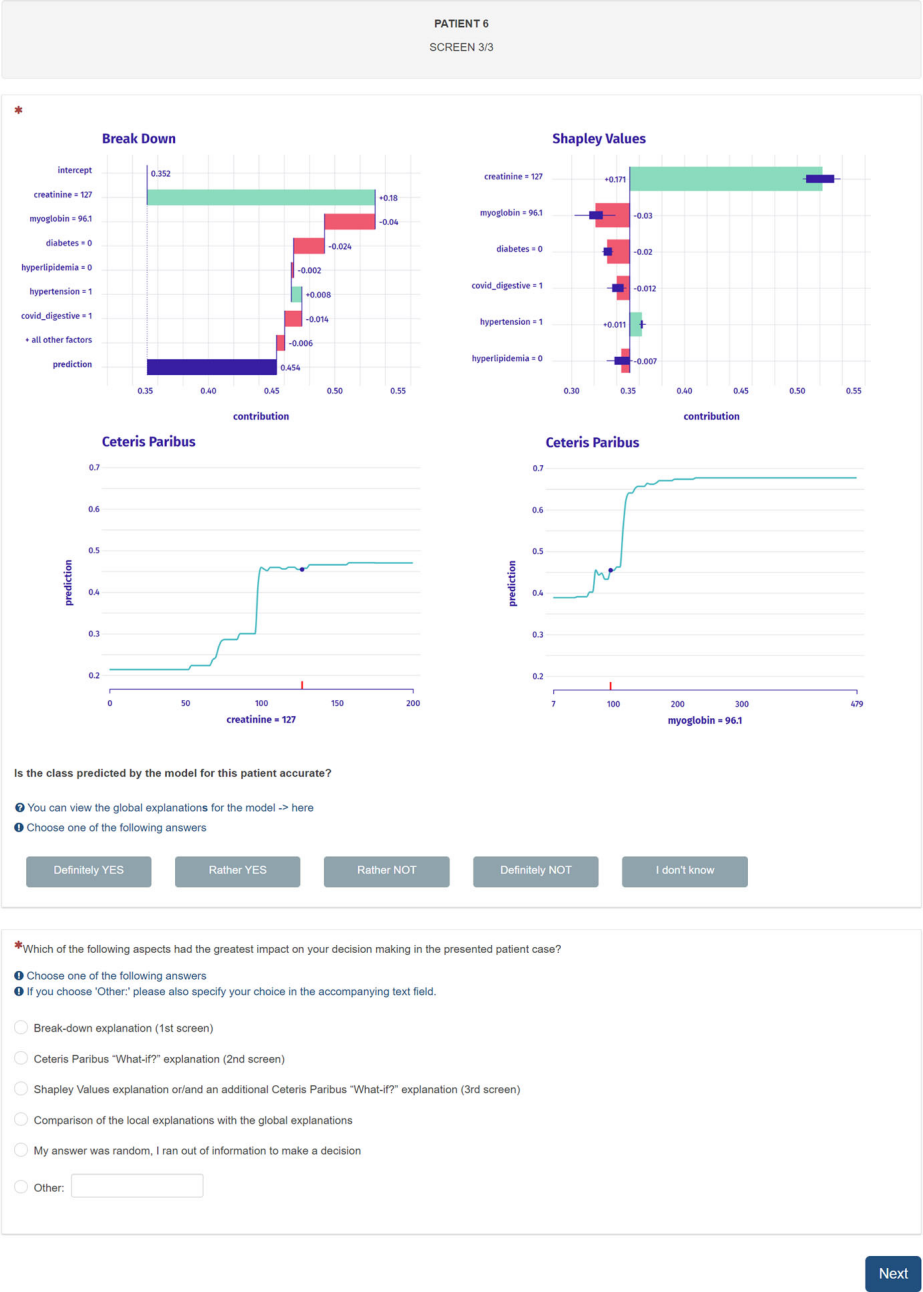
Screenshot from the user study’s questionnaire showing the 3rd screen related to Patient 6 containing a set of four explanations: a Break-down explanations with an additional Ceteris Paribus explanations of the most important variable, and with additional Shapley Values and Ceteris Paribus explanations. At the end of each patient case, we asked for additional input on the most important factor affecting the participant’s decision

These three combinations of evidence were shown *sequentially* so that the participant could change their answer to the pivotal question of class prediction correctness. Note that the results obtained in $Q_1$ serve as a “control group” in our study since we aim to compare them with the results obtained after a sequence $Q_1$–$Q_3$ (see Sect. 2.3 for analogous studies comparing to other baselines). For answers, we chose a 5 point Likert scale consisting of “Definitely/Rather YES/NO” and “I don’t know”. On purpose, half of the presented observations were classified as wrong by the model (6/12). An example of such classification would be when the model predicts a probability of 0.6 while AKI did not occur for this patient. Figure 11 presents the 3rd screen from an exemplary audit process for a single patient. The participant was asked to answer an additional question on the third screen for each patient: “Which of the following aspects had the greatest impact on the decision making in the presented case?” ($Q_4$). The exemplary 1st and 2nd screens for this patient are presented in “Appendix A”.

**Fig. 12:**
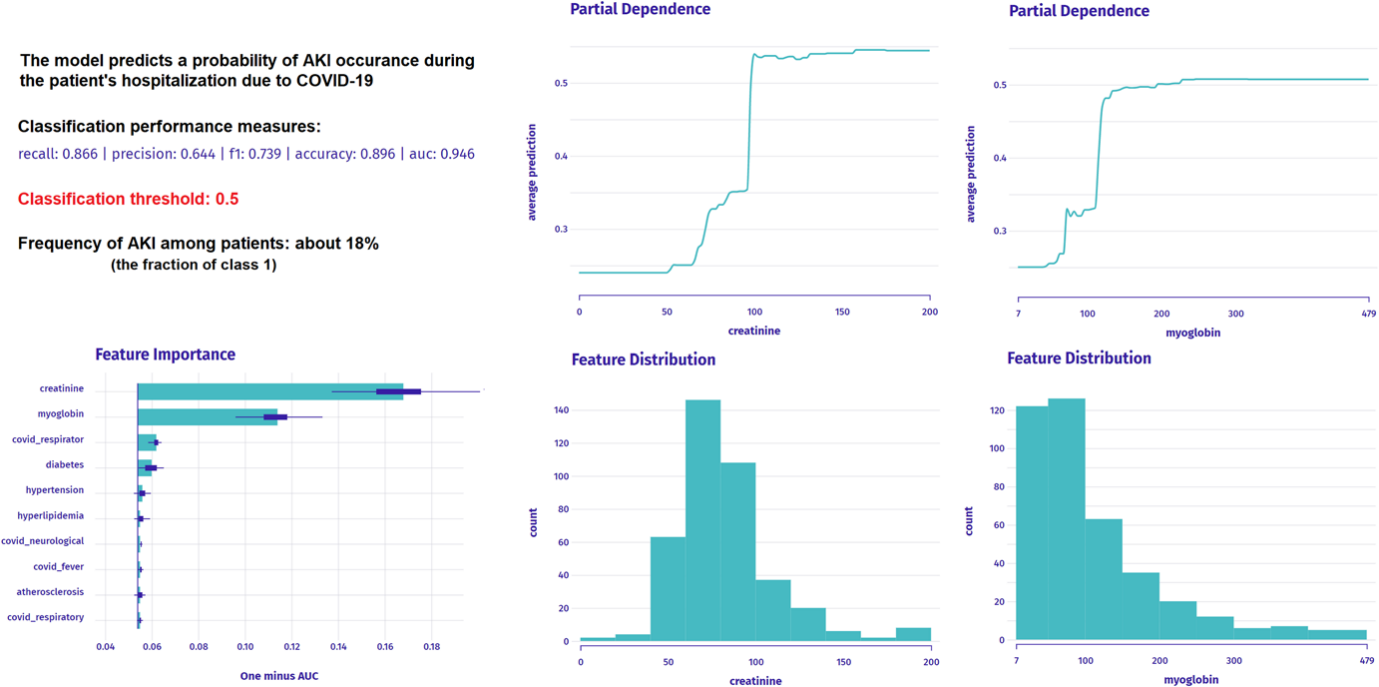
Screenshot from the user study’s questionnaire showing the explanatory context containing global explanations: Permutational Importance, and Partial Dependence explanations of the two most important variables with their distributions. These information were available at all times during the task

To sum up the main task, each participant was tasked with answering 3 sequential questions ($Q_1$–$Q_3$ in Table 4), plus one additional indicating the participant’s thought process ($Q_4$ in Table 5), about 12 patient cases each. Before the main task, the questionnaire made each participant familiar with a broad instruction, which discussed the task, data, model, and explanations, with a particular emphasis on data distributions and global model explanations (Fig. 12). These visualizations were also available to each participant at all times during the questionnaire filling; hence, they are indicated as a possible answer in $Q_4$ for each patient case (shown in Fig. 11). After the main task, there are some additional descriptive questions asked about the process, which allow us to qualitatively analyze the researched phenomenon (Sect. 5.2).

To make sure that the questionnaire is clear, we conducted a *pilot study* in person before the formal study, in which we validated our methodology with 3 participants and took their feedback into account. Additionally, the formal study contained a 13th patient case as a test case before the main task. The study was conducted as a computer-assisted web interview (CAWI) using a professional on-premise software with targeted invitations sent by email.

**Table 4:** Aggregated results from the user study validate our hypotheses

Hypothesis (number of cases = 12)	$Q_1$	$Q_3$	$\Delta Q_3 Q_1$	*P* values
Accuracy increases between $Q_3$ and $Q_1$	${52.2}_{\pm 29.3}$	${65.8}_{\pm 24.2}$	${13.6}_{\pm 11.4}$	0.002; 0.004
Confidence increases between $Q_3$ and $Q_1$	${23.1}_{\pm 13.7}$	${35.3}_{\pm 15.6}$	${12.2}_{\pm 11.8}$	0.004; 0.018
“I don’t know” *decreases* between $Q_3$ and $Q_1$	${12.8}_{\pm 9.8}$	${5.2}_{\pm 5.0}$	${-7.5}_{\pm 7.8}$	0.007; 0.007

We report ${mean}_{\pm sd}$ across the participants’ performance in 12 patient cases, and measure their difference between $Q_3$ and $Q_1$ marked as $\Delta Q_3 Q_1$. We validate each hypothesis with the *t* test and Wilcoxon signed-rank test, hence two *p* values. There is a significant increase in accuracy and confidence between the sequential questions. Additionally, the frequency of ambiguous answers decreases

**Table 5:** Frequency of answers for $Q_4$ averaged across 12 cases times 30 participants

$Q_4$: *Which of the following aspects had the greatest impact on your decision making in the presented patient case?*	
Answer	Frequency (%)
Break-down explanation (1st screen)	16.7
Ceteris Paribus “What-if?” explanation (2nd screen)	27.5
Shapley Values explanation or/and an additional Ceteris Paribus “What-if?” explanation (3rd screen)	35.3
Comparison of the local explanations with the global explanations	19.2
My answer was random, I ran out of information to make a decision	0.5
Other (three descriptive answers in total: a Permutational Importance explanation, both Ceteris Paribus explanations, a high residual value)	0.8

**Fig. 13:**
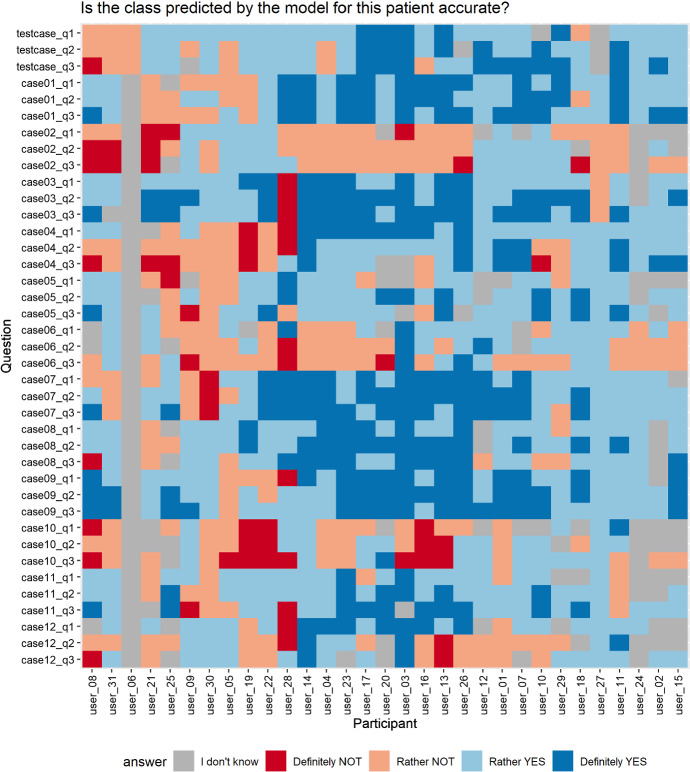
Individual answers in the user study. Rows correspond to consecutive questions. Columns correspond to participants. Colors encode answers. Participants are clustered based on their similarity (Color figure online)

*Participants.* The target population in this study are data science practitioners with varied experience in machine learning and explainability, spanning from machine learning students to scientists researching XIML. Overall, there were 46 answers to our questionnaire, of which 31 were fully completed. Please note that we exclude one of the fully completed answers across reporting the results as it contains an answer of “I don’t know” at each step of the questionnaire, which is rather redundant (see Fig. 13). Thus, we rely on 30 answers in total. Crucially, this user study was anonymous with respect to the participants’ identity, not their origin, as we aim to represent the target population correctly. The questionnaire was concluded with questions related to the participants’ demographic data, e.g. about the participant’s occupation and machine learning experience, which we report in “Appendix B”.

*Expert validity phase to choose proper patient cases.* We conducted an expert validity study on 3 explainable machine learning experts before the described pilot and formal studies. The task was similar; it included answering the main question about the accuracy of model predictions based on information in all of the available explanations for 24 patients from the data (like in $Q_3$). We used the results to unambiguously pick the 12 patient cases where users of the highest expertise most agreed on answers concerning the information carried in explanations. This made the user study less biased with respect to our personal views.

**Fig. 14:**
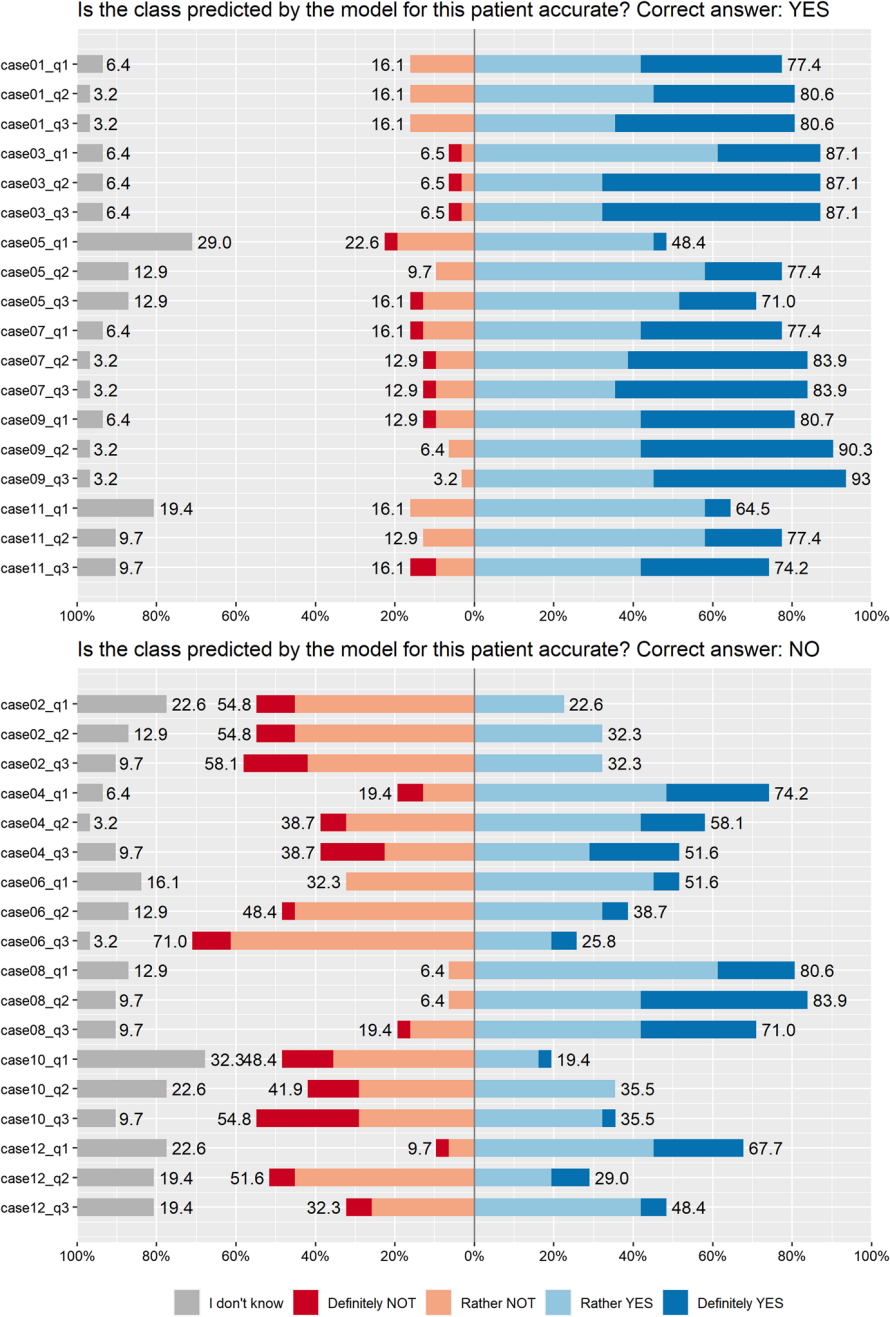
Summary of answers from the main part of the user study. Colors and questions correspond to these presented in Fig. 13. Top panel corresponds to questions related to cases with correct predictions while the bottom panel corresponds to questions with incorrect predictions (Color figure online)

### Quantitative analysis

We first validate the two hypotheses by measuring the performance change between the sequential questions for each patient case using the following statistics:

- Accuracy: frequency of participants choosing “Definitely/Rather YES” when the prediction was accurate and “Definitely/Rather NO” when it was wrong.
- Confidence: frequency of participants choosing “Definitely YES/NO” as oppose to “Rather YES/NO” or “I don’t know”.

Additionally, we validate if the frequency of answers “I don’t know” decreases over the course of questions, which corresponds to increasing human confidence and trust. Table 4 reports the aggregated quantitative results from the user study. We use the *t* test and Wilcoxon signed-rank test to compare the differences $\Delta$ between $Q_3$ and $Q_1$, which serves as a baseline scenario in our study. We omit the analogous results for $Q_2$ where the difference is, as expected, smaller and report detailed numbers for each patient case in Fig. 14 and “Appendix C”.

Since $\Delta Q_3 Q_1$ is positive, the overall conclusion is that the sequential analysis of a model $Q_1$–$Q_3$ with juxtaposing complementary explanations increases both human accuracy and confidence with respect to the single aspect-model explanation $Q_1$. Moreover, Table 5 presents the frequency of answers to $Q_4$ across all cases and participants. We observe an increasing relationship between the impact of consecutive explanations. Participants highlight that in about 19% of cases, juxtaposing global and local explanations had the greatest impact on their decision making, which we also view as a positive outcome towards our thesis.

### Qualitative analysis

At the end of the user study, we asked our participants to share their thoughts on the user study. In the first question, we asked if they saw any positive aspects of presenting a greater number of explanations to the model. This optional question was answered by 19 participants, who most often pointed to the following positive aspects: the greater number of the presented explanations, the more information they obtain (n = 18; 95%), which allows a better understanding of the model (n = 13; 68%), and ultimately increases the certainty of the right decision making (n = 8; 42%) as well as minimizes the risk of making a mistake (n = 2; 11%). Additionally, we asked if the participants identified any potential problems, limitations, threats related to presenting additional model explanations? In 21 people answering this question, the most frequently given answers were: too many explanations require more analysis, which generates the risk of cognitive load (n = 15; 71%), and which may, in consequence, distract the focus on the most important factors (n = 7; 33%). Therefore, some participants highlighted the number of additional explanations as a potential limitation (n = 10; 48%). Moreover, the participants noticed that the explanations must be accompanied by clear instructions for a better understanding of the presented data, because otherwise they do not fulfill their function (n = 6; 29%), and may even introduce additional uncertainty to the assessment of the model (n = 4; 19%).

### Detailed results

**Fig. 15:**
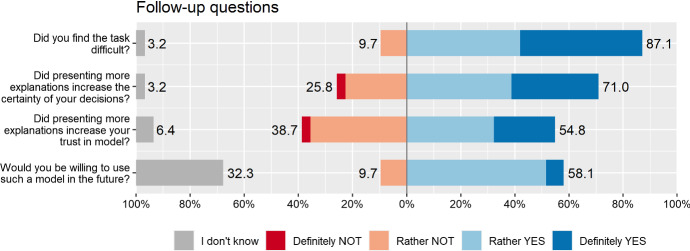
Summary of answers from the follow-up part of the user study

To analyze the results in detail, we deliver the following visualizations. Figure 13 presents specific answers given by the participants at each step of the questionnaire. Participants are clustered based on their answers with hierarchical clustering using the Manhattan distance with complete linkage, which is the best visual result obtained considering several clustering parameters. Note a single gray column corresponding to the removed participant. Overall, looking at the columns, we perceive more and less certain groups of participants, while in rows, we see blocks of three answers of similar color. Figure 14 aggregates the results presented in Fig. 13 and divides them between wrong and accurate predictions. In this example, we better see the characteristic division into blue and red answers, as well as the change in the participants’ certainty over $Q_1$–$Q_2$–$Q_3$. There were some hard cases in our study. Specifically, in case no. 10, participants were on average less accurate in $Q_2$ than in $Q_1$, and in case no. 12, participants were less accurate in $Q_3$ than in $Q_2$. Finally, the user study involved some follow-up questions asked at the end (Fig. 15). The task was rather difficult for the users, from which we deduce that the created test in the form of a user study has high power in a statistical sense. Also, the participants think that presenting more explanations has the potential to increase the certainty and trust in the models.

## Discussion

In this section, we first comment on the user study and then discuss challenges in developing human-centered frameworks for XIML and how it relates to responsible machine learning.

### User study: assumptions, limitations, and future work

Our user study follows the One-Group Pretest-Posttest Design (Shadish et al. 2002; Reichardt 2019), in which we compare the pretest observation, e.g. an answer to $Q_1$, with the posttest observation, e.g. an answer to $Q_2$. Crucially, this allows us to measure the change in human performance across time. Many threats to the internal validity of such a study are least plausible. For example, instrumentation, selection differences, and cyclical changes threats (Reichardt 2019) are non-existent. Further, both the time and maturation effects are directly embedded into the principles of sequential model analysis. Nevertheless, the results like an increase in accuracy and confidence should be interpreted with respect to this experimental design assumption.

Many variables can affect the outcome of such a user study, yet most of them need to be fixed. First, we used only a specific predictive task, a real-world scenario of performing a model audit, and specific sets of explanations, which correspond to the available paths in the grammar of IEMA. To quantify the process and answers at different steps, we constructed a constrained questionnaire containing multiple views instead of allowing the users to interact with the dashboard themselves. In the future, it would be desirable to find ways of measuring a change in human performance when interacting in an open environment. To extend the results, we would like to perform a similar study on another group of stakeholders, e.g. physicians, in the case of predictive tasks concerning medicine. It could also involve other rules from the context-free grammar of IEMA, which correspond to alternative human-model interactions.

When choosing patient cases, we tried to account for a balanced representation of classes and balanced difficulties of predictions. When choosing participants, we aimed to gather answers from machine learning experts as opposed to crowd-sourced laypeople. Considering the above, we constrained the questionnaire to 12 patient cases aiming for about 45 minutes, which we believe allows for a reasonable inference. Overall, participants and results agree with evidence from previous work (Adebayo et al. 2020; Poursabzi-Sangdeh et al. 2021) that finding wrong predictions based on explanations is a difficult task, which, in our view, makes it a more robust evaluation of our methodology. The experiment with human subjects confirmed our hypotheses that juxtaposing complementary explanations increases their usefulness. However, participants raised to attention the *information overload* problem (Poursabzi-Sangdeh et al. 2021)—the quantity of provided information needs to be carefully adjusted so as not to interfere with human decision making.

### Challenges in human-centered explainable and interpretable machine learning

The issues for future human-centered XIML research presented by Choudhury et al. (2020) contain enhancing the technical view in black-box system design through a socio-technical one and lowering the entry threshold for different stakeholders, e.g. domain experts. Specifically, explaining complex predictive models has a high entry threshold, as it may require:

1. **Know-how**: We produce explanations using frameworks that involve high programming skills.
2. **Know-why**: We need to understand the algorithmic part of the model and heavy math behind explanations to reason properly.
3. **Domain knowledge**: We validate explanations against the domain knowledge.
4. **Manual analysis**: We need to approach various aspects of a model and data differently as all valid models are alike, and each wrong model is wrong in its way.

The idea of explainability scenarios introduced by Wolf (2019) may be a starting point for reinforcing our designs by showcasing these requirements. It is possible to enhance the model explanation process to lower the barriers and facilitate the analysis of different model’s aspects. In this section, we introduce three main traits that a modern XIML framework should possess to overcome some of the challenges in the human-model interface.

*Interactivity.* Interactive dashboards are popular in business intelligence tools for data visualization and analysis due to their ease of use and instant feedback loop. Decision-makers can work in an agile manner, avoid producing redundant reports and need less know-how to perform demanding tasks. Unfortunately, this is not the case with XIML tools, where most of the current three-dimensional outputs like colorful plots, highlighted texts, or saliency maps, are mainly targeted at machine learning engineers or field-specialists as oppose to nontechnical users (Miller et al. 2017). As an alternative, we could focus on developing interactive model explanations that might better suit wider audiences. Interactivity in a form of an additional “fourth dimension” helps in the interpretation of raw outputs because users can access more information. Additionally, the experience of using such tools becomes more engaging.

*Customizability.* Interactivity provides an open window for customization of presented pieces of information. In our means, customizability allows modifying the explanations dynamically, which means that all interested parties can freely view and perform the analysis in their way (Sokol and Flach 2020). This trait is essential because human needs may vary over time or be different for different models. With overcoming this challenge, we reassure that calculated XIML outputs can be adequately and compactly served to multiple diverse consumers (Bhatt et al. 2020). Furthermore, looking at only a few potential plots or measures is not enough to grasp the whole picture. They may very well contradict each other or only together suggest evident model behavior; thus, the juxtaposition of model explanations with EDA visualizations is highly beneficial.

*Automation.* A quick feedback loop is desirable in the model development process. However, an endless, manual and laborious model analysis may be a slow and demanding task. For this process to be successful and productive, we have developed fast model debugging methods. By fast, we mean easily reproducible in every iteration of the model development process. While working in an iterable manner, we often reuse our pipelines to explain the model. This task can be fully automated and allow for more active time in interpreting the explanations. Especially in XIML, analyzing the results should take most of the time instead of producing them.

*Dashboard-like XIML frameworks.* Automation and customizability make the framework approachable for diverse stakeholders apparent in the XIML domain. Interactivity allows for a continuous model analysis process. Standard and well-established libraries for model interpretability and explainability documented by Adadi and Berrada (2018) are not entirely going out towards emerging challenges. Although some ideas are discussed by Liu et al. (2017); Hohman et al. (2018), we relate to open-source tools that recently appeared in this area, especially new developments used in machine learning practice. These are mostly dashboard-like XIML frameworks that aim to manifest the introduced traits and parts of the grammar of IEMA (see Table 1).

### Responsiblity in machine learning

Recently, a responsible approach to machine learning is being brought up as a critical factor, and the next step for a successful black-box adoption (Barredo Arrieta et al. 2020; Gill et al. 2020). An interesting proposition concerning model transparency, fairness and security is the Model Cards framework introduced by Mitchell et al. (2019). It aims to provide complete documentation of the model in the form of a short report consisting of various information, e.g. textual descriptions, performance benchmarks, model explanations, and valid context. We acknowledge that apart from the introduced advantages of IEMA and the modelStudio framework, its output serves as a customizable and interactive supplementary resource, generated after model development, for documenting black-box predictive models (Baniecki and Biecek 2021). The idea of responsible and reproducible research is important now more than ever (King 1995; Baker 2016). Roscher et al. (2020) discusses the use of XIML for knowledge discovery, especially in scientific domains. We believe that researchers should be able to easily support their contributions with explanations, which would allow others (especially reviewers) to analyze the model’s reasoning and interpret the findings themselves. For example, the modelStudio framework allows for it through its serverless output, which is simple to produce, save and share as model documentation. The same principle stays for responsible machine learning used in the commercial domain. Decision-making models could have their reasoning put out to the world, making them more transparent for interested parties.

## Conclusion

The topic of explainable machine learning brings much attention recently. However, related work is dominated by contributions with a technical approach to XIML or works focused on providing a list of requirements for its better adoption.

In this paper, we introduce a third way. First, we argue that explaining a single model’s aspect is incomplete. Second, we introduce a taxonomy of explanations that focuses on the needs of different stakeholders apparent in the lifecycle of machine learning models. Third, we describe XIML as an interactive process in which we analyze a sequence of complementary model aspects. Therefore, the appropriate interface for unrestricted model analysis must adopt interactivity, customization, and automation as the main traits. The introduced grammar of Interactive Explanatory Model Analysis has been designed to effectively adopt a human-centered approach to XIML. Its practical implementation is available through the open-source modelStudio framework. To our knowledge, this is the first paper to formalize the process of interactive model analysis.

We conducted a user study to evaluate the usefulness of IEMA, which indicates that an interactive sequential analysis of a model may increase the accuracy and confidence of human decision making. The grammar of IEMA is founded on related work and our research neighbourhood’s experiences in the explanatory analysis of black-box machine learning predictive models. The domain-specific observations might influence both practical and theoretical insight; thus, in the future, we would like to perform more human-centric experiments to study how possibly unidentified stakeholders analyze models.

## Figures and Tables

**Fig. 16:**
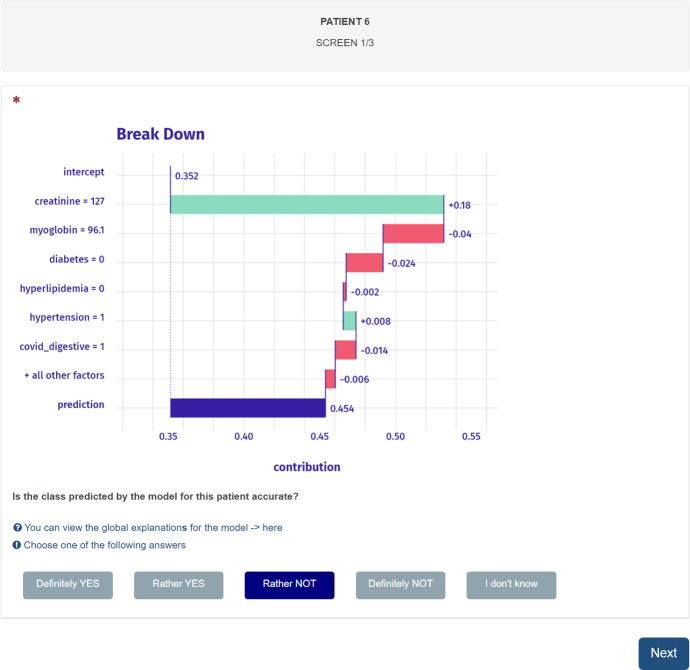
Screenshot from the user study’s questionnaire showing the 1st screen containing a single Break-down explanation related to Patient 6

**Fig. 17:**
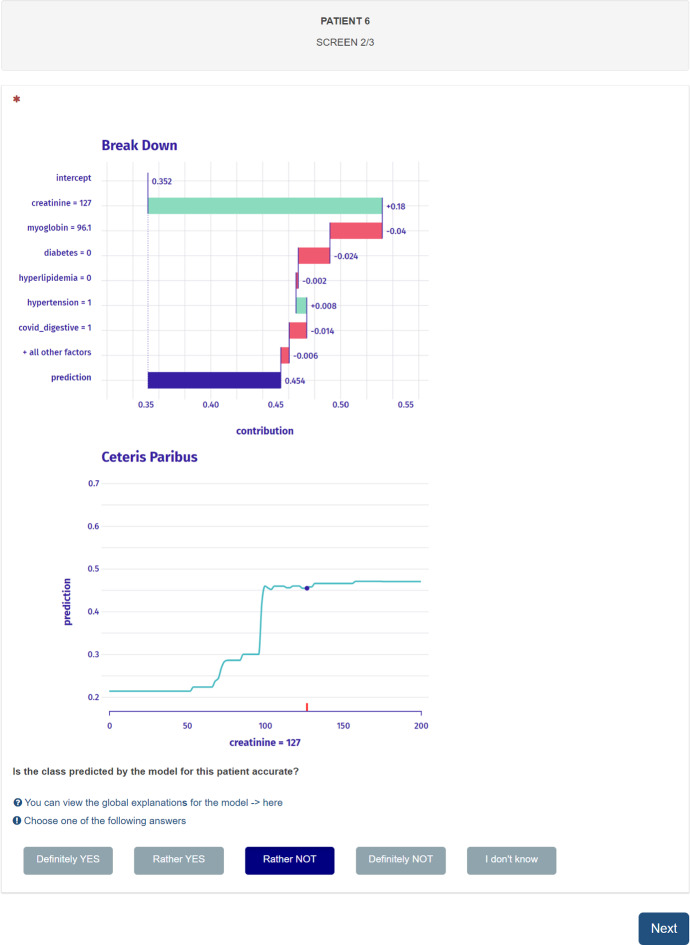
Screenshot from the user study’s questionnaire showing the 2nd screen containing a Break-down explanation with an additional Ceteris Paribus explanation of the most important variable

## A User study: screenshots of the questionnaire

See Figs. 16 and 17.

## B User study: demographic profile of participants

See Table 6.

**Table 6:** The demographic profile of 31 participants who fully answered the questionnaire in the user study

	Count	Frequency (%)
*Gender*		
Man	25	80.6
Woman	6	19.4
I don’t want to answer	0	0
*What year of study are you in?*		
3rd year (BSc)	17	54.8
4th year (BSc & MSc)	7	22.6
5th year (MSc)	3	9.7
Other, e.g. PhD	4	12.9
*How much experience do you have in machine learning?*		
1 (No experience)	0	0.0
2	4	12.9
3	8	25.8
4	7	22.6
5	7	22.6
6 (Extensive experience)	5	16.1
*How much experience do you have in explainable machine learning?*		
1 (No experience)	4	12.9
2	6	19.4
3	10	32.3
4	6	19.4
5	3	9.7
6 (Extensive experience)	2	6.5
*How much experience do you have in using machine learning in medical applications?*		
No experience	9	29.0
Participation in one project	18	58.1
Multiple projects and/or collaboration with medical staff	4	12.9

## C User study: detailed quantitative results

See Tables 7 and 8.

**Table 7:** Accuracy for each patient case measured across the answers of 30 participants

Case no.	$Q_1$	$\Delta Q_2 Q_1$	$Q_2$	$\Delta Q_3 Q_2$	$Q_3$	$\Delta Q_3 Q_1$
01	80.0	$+$ 3.3	83.3	0.0	83.3	$+$ 3.3
02	56.7	0.0	56.7	$+$ 3.3	60.0	$+$ 3.3
03	90.0	0.0	90.0	0.0	90.0	0.0
04	20.0	$+$ 20.0	40.0	0.0	40.0	$+$ 20.0
05	50.0	$+$ 30.0	80.0	$-$ 6.7	73.3	$+$ 23.3
06	33.3	$+$ 16.7	50.0	$+$ 23.3	73.3	$+$ 40.0
07	80.0	$+$ 6.7	86.7	0.0	86.7	$+$ 6.7
08	6.7	0.0	6.7	$+$ 13.3	20.0	$+$ 13.3
09	83.3	$+$ 10.0	93.3	$+$ 3.3	96.7	$+$ 13.3
10	50.0	$-$ 6.7	43.3	$+$ 13.3	56.7	$+$ 6.7
11	66.7	$+$ 13.3	80.0	$-$ 3.3	76.7	$+$ 10.0
12	10.0	$+$ 43.3	53.3	$-$ 20.0	33.3	$+$ 23.3
**Mean**	**52.2**	$+$ 11.4	63.6	$+$ 2.2	**65.8**	$+$ **13.6**
**SD**	**29.3**	14.4	26.3	10.9	**24.2**	**11.4**
**Median**	53.3	$+$ 8.3	68.3	0.0	73.3	$+$ 11.7

$\Delta Q_3 Q_1$ indicates the difference in accuracy between $Q_3$ and $Q_1$. In bold, we highlight the results reported in Table 4

**Table 8:** Confidence for each patient case measured across the answers of 30 participants

Case no.	$Q_1$	$\Delta Q_2 Q_1$	$Q_2$	$\Delta Q_3 Q_2$	$Q_3$	$\Delta Q_3 Q_1$
01	36.7	0.0	36.7	$+$ 10.0	46.7	$+$ 10.0
02	10.0	0.0	10.0	$+$ 6.7	16.7	$+$ 6.7
03	30.0	$+$ 30.0	60.0	0.0	60.0	$+$ 30.0
04	33.3	$-$ 10.0	23.3	$+$ 16.7	40.0	$+$ 6.7
05	6.7	$+$ 13.3	20.0	$+$ 3.3	23.3	$+$ 16.7
06	6.7	$+$ 3.3	10.0	$+$ 6.7	16.7	$+$ 10.0
07	40.0	$+$ 10.0	50.0	$+$ 3.3	53.3	$+$ 13.3
08	20.0	$+$ 23.3	43.3	$-$ 10.0	33.3	$+$ 13.3
09	43.3	$+$ 6.7	50.0	0.0	50.0	$+$ 6.7
10	16.7	$-$ 3.3	13.3	$+$ 16.7	30.0	$+$ 13.3
11	6.7	$+$ 13.3	20.0	$+$ 20.0	40.0	$+$ 33.3
12	26.7	$-$ 10.0	16.7	$-$ 3.3	13.3	$-$ 13.3
**Mean**	**23.1**	$+$ 6.4	29.4	$+$ 5.8	**35.3**	$+$ **12.2**
**SD**	**13.7**	12.3	17.6	8.9	**15.6**	**11.8**
**Median**	23.3	$+$ 5.0	21.7	$+$ 5.0	36.7	$+$ 11.7

$\Delta Q_3 Q_1$ indicates the difference in confidence between $Q_3$ and $Q_1$. In bold, we highlight the results reported in Table 4

## Footnotes

1 Cristiano Ronaldo is one of the most famous footballers globally; hence, variables attributing to his worth may be of high interest.

2 The modelStudio dashboard for the FIFA-20 use-case: https://iema.drwhy.ai.

3 The modelStudio dashboard for the COVID-19 use-case: https://rai-covid.drwhy.ai.
